# Building climate-resilient health systems in Sierra Leone: addressing the dual burden of infectious and climate-related diseases

**DOI:** 10.1186/s40249-025-01294-9

**Published:** 2025-03-24

**Authors:** Umaru Sesay, Augustus Osborne

**Affiliations:** 1 Sierra Leone Field Epidemiology Training Program, National Public Health Agency, Freetown, Sierra Leone; 2 Institute for Development, Freetown, Sierra Leone

**Keywords:** Climate change, Health system, Emergency, Health Policy, Public Health, Global Health, Sierra Leone

## Abstract

Climate change presents a profound challenge to global health, disproportionately affecting low-income countries like Sierra Leone. This opinion examines the dual burden of infectious and climate-related diseases and their implications for Sierra Leone’s health systems. Rising temperatures and changing rainfall patterns intensify vector-borne diseases such as malaria and Lassa fever, while flooding exacerbates waterborne diseases in overcrowded urban areas. Concurrently, climate-driven food insecurity worsens malnutrition, particularly among children, and heat stress contributes to the growing prevalence of non-communicable diseases. These overlapping health crises strain Sierra Leone’s fragile health system, characterized by inadequate infrastructure, workforce shortages, weak surveillance systems, and limited financial resources. The dual disease burden not only increases morbidity and mortality but also deepens existing health inequities and inequalities. To address these challenges, this opinion underscores the need for climate-resilient health policies and systems. Key recommendations include strengthening healthcare infrastructure, building workforce capacity through targeted training, fostering community-based adaptation strategies, and enhancing international collaboration and financing. Establishing robust research and data systems is also critical to monitor and mitigate climate-related health impacts. By prioritizing response to dual burden of infectious and climate-related diseases within health policy frameworks, Sierra Leone can build a resilient health system that safeguards public health and promotes sustainable development.

## Background

Climate change, driven by anthropogenic and natural factors, represents a significant global public health threat, with far-reaching impacts across multiple sectors, including health systems [1–3]. This challenge is particularly acute in low-income countries like Sierra Leone, where limited resources constrain the capacity to effectively address this pressing public health issue [4]. Currently, seven out of the ten countries with the greatest vulnerability to climate change globally are located in Africa [5], underscoring the susceptibility of populations to a wide range of climate-sensitive health challenges, including vector-borne, waterborne, and heat-related illnesses. These health threats are compounded by Sierra Leone’s geographic vulnerabilities, socioeconomic challenges, and fragile health systems.

In April 2024, Sierra Leone established a climate unit within the Ministry of Health to coordinate efforts to reduce the impact of climate change on health [6]. Despite this initiative, the country remains among the top 10% globally most vulnerable to climate change [7]. While the Sierra Leone government, through the Ministry of Health, has made strides in developing policies and frameworks such as the National Climate Change Strategy and Action Plan (2015) and the National Climate Change Policy Framework [8], there remains a substantial gap in implementing these frameworks, especially at the district and community levels.

A critical shortcoming of the existing frameworks is the insufficient integration of climate change considerations into the health system, which is essential for effective response interventions [8]. Environmental challenges, including deforestation, bush burning, and the emission of hazardous chemicals from industrial activities, exacerbate climate-related risks in Sierra Leone [9]. Additionally, factors such as inadequate healthcare investment, low socioeconomic status, geographic location, rapid urbanization, and recurring public health emergencies including civil war, the Ebola outbreak, and the COVID-19 pandemic have compounded the country’s vulnerability to the health impacts of climate change [8].

The adverse effects of climate change have disrupted Sierra Leone’s natural ecosystems, contributing to rising temperatures, sea level rise, coastal erosion, erratic rainfall patterns, and frequent flooding [7]. These environmental changes have resulted in a wide array of health-related challenges. Rising temperatures and changing rainfall patterns have intensified vector-borne diseases, such as malaria and Lassa fever, while flooding has increased waterborne diseases, such as cholera and diarrhoea, particularly in overcrowded urban areas [2]. Prolonged heatwaves and rising temperatures have also led to heat stress and exacerbated non-communicable diseases (NCD), such as cardiovascular and respiratory illnesses [2]. Furthermore, climate-driven food insecurity has worsened malnutrition, particularly among children.

Studies have shown variations in climatic factors, such as precipitation, temperature, and humidity, significantly influence the patterns and prevalence of infectious and climate-related diseases [10–14]. Addressing these challenges requires building a resilient health system capable of responding effectively to the dual burden of infectious and climate-related diseases. This is critical to enhancing the efficiency of Sierra Leone’s healthcare system and fostering progress towards achieving the Sustainable Development Goals (SDGs) and Universal Health Coverage (UHC).

This opinion aims to explore the impact of climate change on health systems in Sierra Leone, with a particular focus on the dual disease burden of infectious and climate-related diseases. Additionally, it outlines policy recommendations to strengthen climate-resilient health systems in Sierra Leone, emphasizing multisectoral collaboration, community engagement, and international partnerships.

## Non-climate-related health system issues

Sierra Leone faces substantial challenges in delivering efficient healthcare services to its population. Higher healthcare costs compounded with a limited trained health workforce and low motivation, inadequate infrastructure, belief in traditional medicines, and disruption of healthcare services by outbreaks affect health service delivery. Other health system issues include corruption, lack of accountability; and minimal inclusion of community stakeholders in health policy development. This lack of stakeholder engagement can result in policies that do not adequately reflect the needs and priorities of the population, limiting their effectiveness and sustainability.

## Impact of climate change on health in Sierra Leone

### Rising temperatures and vector-borne and zoonotic diseases

**Fig. 1:**
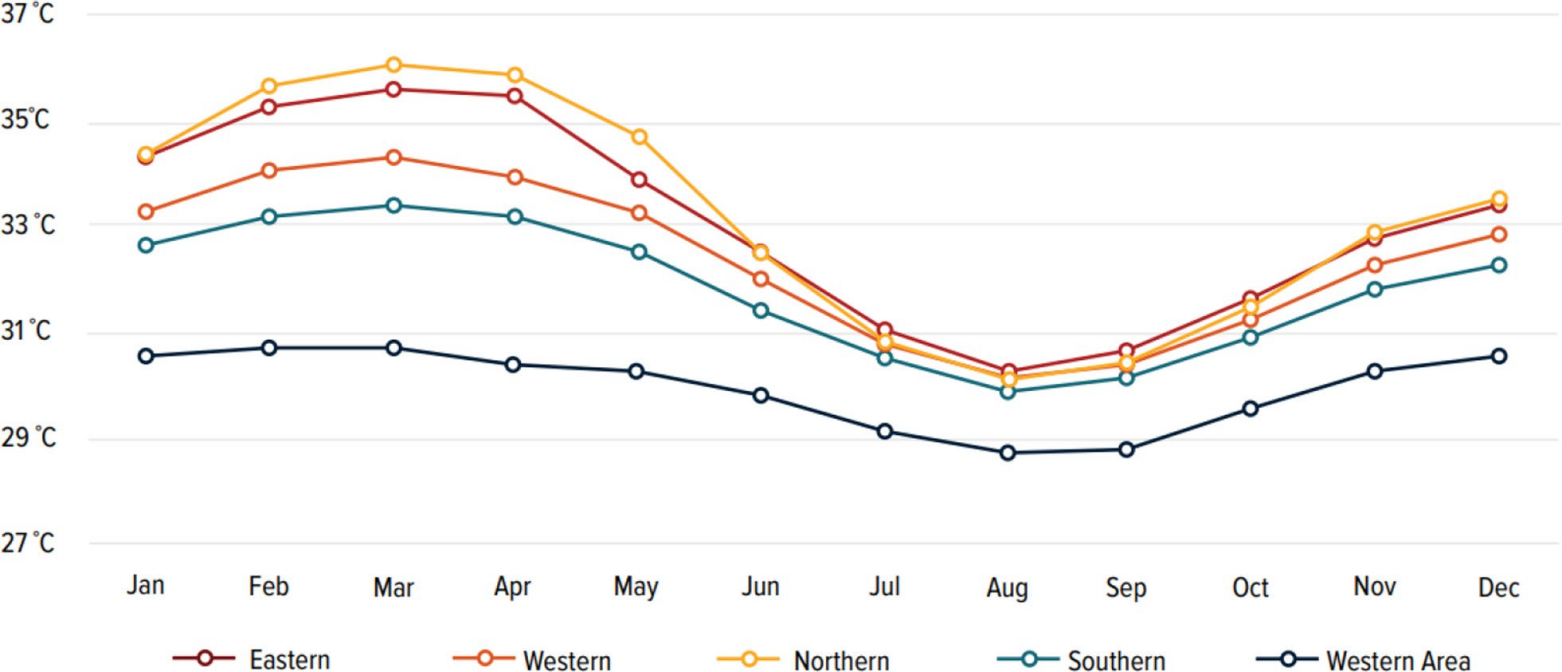
Average daily maximum temperature across Sierra Leone under representative concentration pathway 8.5 during the 2050s. Source: World Bank Climate Change Knowledge Portal

Rising temperature is a key driver of climate change, increasing the risk of vector-borne and zoonotic diseases, which already threaten Sierra Leone [11]. The country struggles to manage diseases like malaria, Lassa fever, snakebite, and diarrhea. While rising temperatures create conditions more conducive to the spread of diseases, it is important to note that the increase in malaria cases cannot be attributed solely to this factor. Malaria, for instance, accounts for 38% of hospital admissions and 50% of outpatient cases [15], but its prevalence is influenced by a combination of factors, including environmental, socioeconomic, and healthcare-related challenges. This high disease burden places immense pressure on the healthcare system, which is already strained. Additionally, Lassa fever leads to 20% hospitalization and more than 90% fatality among pregnant women in their third trimester [16]. These statistics highlight the urgent need for effective surveillance and intervention strategies to mitigate the impacts of these diseases. The temperature rise not only increases morbidities and mortalities, but it also increases the vicious cycle of poverty. A recent report reveals that Sierra Leone’s average annual temperature has risen by 0.8 °C over the last five decades, with monthly maximum temperatures averaging about 30 °C and showing little fluctuation across provinces [17]. Forecasts predict that by 2030, the annual temperature will increase by an additional 1.1 °C, with a further rise of 2.2 °C anticipated by 2050. This anticipated projection is way above the conference of the parties 26 agreement target of not more than 1.5 °C [18]. The most pronounced temperature and heat index increases are expected in the Northern and Eastern provinces, whereas the Southern and Western provinces are likely to experience comparatively smaller changes (Fig. 1) [17]. The anticipated temperature rise in Sierra Leone, especially in the Northern and Eastern regions is alarming, given their vulnerability to socioeconomic challenges and weak healthcare systems. Notably, the Eastern region is a known endemic region for Lassa fever in Sierra Leone [19]. The projected temperature increase is especially concerning, as higher temperatures enhance the interaction of certain vectors like *Mastomys natalensis* (the reservoir host of Lassa fever) with humans. These challenges hinder progress toward SDG targets, particularly those related to health (SDG 3), poverty reduction (SDG 1), and climate action (SDG 13). The low economic activity also affects revenue generation, thereby affecting healthcare service utilization, especially for low-income families. Thus, these factors underscore the need for integrated public health and environmental interventions to mitigate the impact of climate change on vector-borne diseases and rural livelihoods.

### Flooding and waterborne diseases

Over the years, flooding has become a major public health issue in Sierra Leone, causing significant destruction to lives and properties. Between 2012 and 2017, Sierra Leone experienced devastating flooding events, affecting thousands of people and causing hundreds of deaths [20]. These disasters caused immediate loss of life and lasting impacts on infrastructure, livelihoods, and health outcomes. Despite the severe consequences associated with flooding, Sierra Leone currently lacks a coordinated structure for responding to flooding and other disasters, exacerbating the impact of these occurrences. This absence of a comprehensive disaster management framework exacerbates flooding occurrence, hindering timely and efficient responses that could mitigate damage and protect vulnerable populations. The lack of preparedness and response mechanisms means that communities are often left to fend for themselves in the aftermath of flooding, leading to increased suffering and loss. Frequent flooding events create favorable breeding environments for vectors like *Anopheles* mosquitoes, enhancing their population growth and efficiency in transmitting diseases. Additionally, flooding provides ideal conditions for the proliferation of other water-borne and vector-borne diseases, including cholera (*Vibrio cholerae*), bacillary dysentery (*Shigella* species), and amoebic dysentery (*Entamoeba histolytica*). Another critical factor contributing to the rise in water-borne diseases is the lack of access to safe drinking water. In 2019, 58% of the population did not have access to a reliable and safe water supply [21]. This issue is compounded by overpopulation, inadequate infrastructure, weak governance, and widespread poverty [22, 23]. Consequently, Sierra Leone remains one of the countries with the highest burden of water-borne diseases globally [24]. As Sierra Leone remains vulnerable to climate change, the increasing burden of vector- and water-borne diseases resulting from flooding events exacerbated by climate change threatens to overwhelm the country's healthcare infrastructure. This strain undermines the healthcare system's capacity to deliver equitable and accessible care, a critical objective of UHC. Vulnerable populations, particularly those residing in urban slums and rural areas, bear the brunt of these challenges, facing heightened risks that exacerbate health inequities and hinder progress toward SDG 3. Furthermore, the aftermath of flooding marked by the destruction of livelihoods and critical infrastructure perpetuates cycles of poverty (SDG 1) and stifles economic growth (SDG 8), compounding the long-term socioeconomic and health impacts on the population.

### Food insecurity and malnutrition

Food production in Sierra Leone largely depends on rainfall. However, climate pattern shifts have recently reduced productivity, exacerbating food insecurity. In 2022, 81% of households faced food insecurity, with 14.9% in extreme conditions [25]. Malnutrition, driven by food insecurity, remains a critical public health issue in Sierra Leone, with 26% of the population affected by stunting and 5% wasted [26]. As acute malnutrition can lead to severe health complications and increased mortality risk, these statistics underscore the urgent need for interventions to address nutritional deficiencies and improve food access. Low agricultural output and limited access to nutritious food weaken immune systems, particularly in children under five, increasing illness and mortality rates. Thus, conditions resulting from malnutrition will further increase the disease burden, thereby overwhelming the already fragile health system. A critical factor affecting food production in Sierra Leone is the inconsistent patterns of rainfall, driven by man-made activities like deforestation, and the improper deposition of waste such as chemical waste in water bodies and lands. Consequently, this affects the growth of aquatic animals like fish, and crop production on land. As malnutrition contributes to a growing disease burden, the healthcare system faces overwhelming challenges. The interplay between food insecurity and health outcomes necessitates a comprehensive approach that includes enhancing agricultural resilience, improving food distribution networks, and implementing nutrition-focused health interventions. Without urgent action, vulnerable populations particularly children under five and those residing in rural areas will remain disproportionately affected, further exacerbating health disparities and impeding progress toward SDG 3, as well as increasing the vicious cycle of poverty, SDG 1.

### Heat stress and non-communicable diseases

Rising temperatures in Sierra Leone are driving heat stress and a surge in non-communicable and respiratory diseases, posing significant public health issues. The country's healthcare system struggles to respond due to limited resources, insufficiently trained personnel, inadequate infrastructure, and low public awareness. A significant factor in this crisis is the absence of a comprehensive framework to tackle NCDs exacerbated by climate change. The government of Sierra Leone prioritizes combating infectious diseases due to the country’s history of epidemics [27]. However, this focus sidelines the growing burden of NCDs and heat stress-related conditions. Limited investment in NCD prevention, diagnosis, and care worsens outcomes, delaying treatment and increasing mortality rates. Overlooking the rising risks of heat stress and NCDs is alarming, as Sierra Leone already faces the heavy burden of communicable diseases. This dual strain threatens to overwhelm its resource-limited healthcare system, fuelling poor health outcomes, reduced productivity, and hindering national development. Limited knowledge on the benefits of physical exercise, poor balance diet practice, and poor healthcare-seeking behaviour are drivers exacerbating NCDs in Sierra Leone. Strengthening healthcare systems, prioritizing NCD prevention and management, and rolling out public education campaigns are vital. In the midst of the projected climatic conditions, failure to address it urgently, increases heat stress and NCDs that will overwhelm the fragile healthcare system. Ultimately, this will undermine the country’s ability to provide equitable and accessible care, a core objective of UHC. Vulnerable populations, particularly those with limited access to healthcare and education, are disproportionately affected, further widening health disparities and challenging SDG 3. Poor health outcomes stemming from NCDs and heat stress reduce workforce productivity, hinder economic growth (SDG 8), and perpetuate poverty (SDG 1). The health impacts of rising temperatures highlight the need for climate adaptation strategies, aligning with SDG 13.

### Sea level rise

**Fig. 2:**
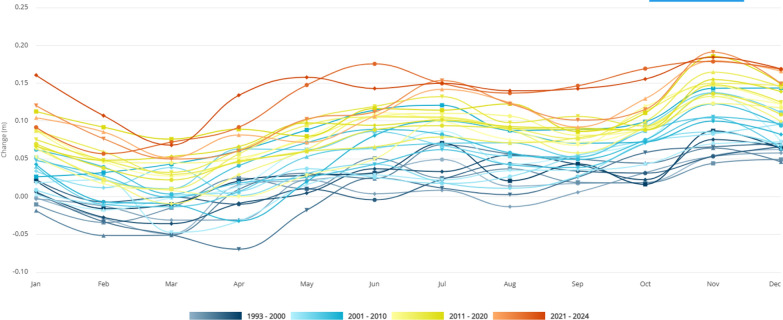
Total sea level change by month (1993–2024)*.* Source: World Bank Climate Change Knowledge Portal [28]

With approximately 2 million Sierra Leoneans living along the coastline, the ongoing rise in sea levels poses an escalating threat to public health and safety (Fig. 2). The absence of a structured framework or tools to monitor the impacts of climate change-related sea level rise exacerbates this risk [17]. The damage triggered by rising sea levels risks not only loss of lives and property but also the displacement of entire communities, an escalation in poverty, disruptions to agriculture, and a decline in water quality. As the nation struggles to meet necessities like food, water, and shelter, strengthening systems to address and reduce the effects of rising sea levels due to climate change becomes an urgent priority. This includes investing in monitoring systems to understand and anticipate changes, implementing adaptive measures to protect vulnerable communities, and promoting sustainable practices to build resilience against future climate-related challenges. Failure to enact decisive control actions, rising sea levels and its associated impacts, will place additional pressure on the healthcare system, undermining efforts to achieve UHC. Vulnerable coastal populations face disproportionate risks, exacerbating health disparities and hindering progress toward SDG 3 and SDG 10. Displacement, poverty, and disruptions to agriculture and livelihoods undermine economic stability, impeding progress toward SDG 1 and SDG 8. The lack of monitoring systems and adaptive measures limits Sierra Leone’s ability to build resilience against climate-related challenges, hindering progress toward SDG 13.

## Health system challenges in Sierra Leone

Sierra Leone, one of the poorest nations globally, struggles with significant obstacles such as insufficient infrastructure, inadequate equipment, a shortage of skilled personnel, weak disease monitoring systems, and limited finance. These challenges greatly hinder the delivery of healthcare services and adversely impact the health outcomes of its population.

### Inadequate infrastructure

Although access to healthcare infrastructure is estimated to be within 3 km between health centers [29], this distance is often greater in rural areas, creating substantial barriers to care for those living in remote communities. In urban areas, healthcare facilities are more accessible but overwhelmed by excessive caseloads. Providing healthcare to address emerging public health issues, such as heat stress and respiratory conditions driven by climate factors, remains a significant challenge. To date, there has been minimal collaboration between the private sector and the government in strengthening healthcare infrastructure and equipping facilities to respond to these emerging public health threats. Private sector involvement is crucial in addressing these gaps by providing financial investment, technical expertise, and innovative solutions to enhance healthcare delivery. However, the lack of strong public–private partnerships has hindered progress in building resilient healthcare systems capable of addressing existing and emerging health challenges.

### Workforce shortages

Sierra Leone has one of the lowest healthcare worker-to-population ratios globally, with only 1.7 nurses and 0.2 physicians serving every 10,000 people [30]. This shortage is further compounded by the unequal distribution of healthcare workers, with the majority residing in urban areas, leaving rural populations disadvantaged in accessing essential healthcare services. The migration of healthcare workers seeking better opportunities in high-income countries further exacerbates this shortage, creating a critical gap in the availability of skilled personnel across the country. Public health emergencies place significant pressure on the healthcare workforce, frequently pulling limited staff away from other essential health priorities, including those associated with climate-related conditions. Furthermore, a lack of adequate capacity-building to tackle climate-related health challenges leaves the system ill-prepared, increasing the likelihood of severe illnesses and fatalities. Beyond workforce challenges, Sierra Leone faces a significant barrier in addressing climate-related health issues due to the lack of community-based frameworks. Unlike infectious diseases, where community health workers play a vital role in detection and care, no such systems exist for managing climate-induced health impacts, hindering the country’s response to climate change.

### Weak disease surveillance systems

Sierra Leone's public health surveillance system has demonstrated remarkable resilience and evolution despite experiencing severe disruptions during multiple crises: the civil conflict (1991−2002), the Ebola outbreak (2014−2015), and the COVID-19 pandemic. The country has advanced in disease surveillance but faces persistent challenges in detecting, analysing, interpreting, and reporting public health events. The limitations in human resources, logistical support, and financial capacity significantly impede the effective operation of the surveillance system, compromising its ability to provide timely and accurate public health intelligence. Additionally, a critical gap in Sierra Leone's surveillance framework has emerged from its historical focus on infectious diseases over the past decades. This prioritization has led to the relative neglect of NCD surveillance, creating a significant blind spot in the nation's public health monitoring capabilities. This imbalance has become particularly problematic as the country faces emerging challenges from climate change-related health impacts, often manifest as or exacerbating NCDs.

### Financial constraints

Sierra Leone, a low-resource country with a fragile healthcare system, allocated merely 5.2% of its government budget to healthcare by the end of 2022 [31], falling below the 15% target established by the Abuja Declaration [32]. This inadequate funding severely constrains the health system's capacity to provide efficient healthcare services, particularly in addressing emerging health challenges associated with climate change. The current healthcare financing structure in Sierra Leone line itemised-budgeting demonstrates patterns of unsustainability. Out-of-pocket payments constitute the majority of healthcare revenue (52.7%), followed by donor funding (28%) for healthcare spending by the end of 2022 [31]. This heavy reliance on out-of-pocket payments presents a significant barrier to healthcare access, particularly affecting the most vulnerable populations. This financial burden becomes especially problematic for climate-related health conditions, where the lack of dedicated funding mechanisms compounds the challenges of seeking and receiving appropriate medical care. The situation creates a paradox: as climate-related health impacts increase, the financial barriers to accessing healthcare services may lead to delayed treatment-seeking behaviour and potentially worse health outcomes. This financing landscape underscores the urgent need for sustainable healthcare funding reforms in Sierra Leone, particularly as the country faces emerging health challenges from climate change. Without substantial increases in government healthcare funding and the development of more equitable financing mechanisms, the health system's capacity to respond to both current and future health challenges, including those related to climate change, will remain severely compromised.

## Policy recommendations for building climate-resilient health systems

As Sierra Leone struggles to rebuild its healthcare system, addressing the impacts of climate change is critical for the effectiveness and performance of the healthcare system. The following policy recommendations are as follows:

### Climate-resilient infrastructure

The strengthening of existing healthcare infrastructure is a critical priority for enhancing the capacity to address climate-related health conditions in Sierra Leone. This improvement requires a comprehensive approach to resource allocation, provision of equipment, and, where necessary, the construction of additional healthcare facilities, particularly in underserved rural areas. To achieve these objectives, the government must cultivate robust public–private partnerships and actively engage with the private sector to leverage financial and technical support for climate change response. The success of these initiatives hinges on sustained commitment from government and private sector stakeholders. Establishing clear mechanisms for collaboration and accountability will be essential in building a more resilient healthcare system capable of effectively addressing the growing burden of climate-related health conditions.

### Strengthening workforce capacity

The lack of expertise among healthcare professionals in managing climate-related health diseases poses a critical weakness within Sierra Leone’s healthcare response systems. Bridging this knowledge deficit demands swift action through extensive capacity-building initiatives to address these emerging health concerns. A comprehensive strategy for workforce development involves implementing several key measures. First, stakeholders should establish well-structured in-service training programs for current healthcare workers, concentrating on assessing and managing climate-associated health diseases. At the same time, medical education institutions should be encouraged to introduce modules on climate change and its health implications into their standard curricula, ensuring future professionals are well-equipped to address these pressing challenges. Second, community health workers should receive targeted training in identifying and addressing climate-related health diseases or conditions. This training should prioritize early symptom recognition, appropriate referral systems, and improved communication with higher-level care facilities. Last, stakeholders should devise robust incentive schemes for healthcare personnel working in remote regions supporting climate change response. These incentives must be backed by developing climate change policy frameworks that address health worker migration drivers.

### Community-based adaptation

Engaging communities is crucial for reducing the effects of climate change on the health system, given that everyday actions within these groups play a substantial role in this global challenge. The active involvement of individuals in community-based climate initiatives forms a cornerstone of impactful response strategies. To achieve this, stakeholders must focus on fostering sustainable habits and promoting behavioural shifts at the community level on climate change. This entails adopting a holistic approach that inspires individuals to embrace environmentally conscious actions and contribute to positive ecological outcomes. Key avenues for community participation include:

**Education and awareness**: Raising awareness about the effects of climate change on public health and healthcare systems is essential. By enhancing understanding, individuals are more likely to adopt preventive measures that mitigate the impacts of climate change. This proactive approach can lead to lower exposure to climate change-related health risks.

**Environmental management**: Encouraging initiatives like effective waste management and tree planting is crucial in addressing climate risks such as heat stress and flooding. These actions help alleviate pressure on an overstretched healthcare system, enhancing public health outcomes.

**Community-driven monitoring**: Developing systems for local monitoring of environmental shifts is essential for strengthening community resilience in climate change initiatives. This localized oversight can significantly enhance health outcomes and reinforce the effectiveness of community health systems.

By prioritizing community education and active engagement in climate solutions, we can enhance the effectiveness of mitigation efforts, thereby contributing to better health and environmental resilience at the local level.

### International support and financing

Building climate-resilient health systems demands considerable financial investments and the establishment of sustainable funding frameworks. In light of the current limitations in healthcare financing within Sierra Leone, there is an urgent need for a significant boost in funding to mitigate climate-related health challenges and fortify the resilience of the country’s health infrastructure. To achieve sustainable healthcare funding with a climate-sensitive approach, we propose the following key actions:

**Strengthen partnerships**: Collaborate with international and local health development organizations to mobilize financial resources for climate-resilient health programs [33].

**Encourage innovative revenue streams**: Create new, sustainable sources of funding by introducing mechanisms like levies on entities or practices that contribute to environmental harm and expanding health insurance coverage systems for health system response to climate change.

**Boost health financing**: Allocate a larger share of government revenue to health, striving to meet the Abuja Declaration funding targets while ensuring climate change adaptations are embedded in these plans. Also, industrial institutions or organizations that emit larger waste into the community pay additional taxes as a form of compensation to the population [34].

**Develop a climate-health emergency fund**: Urge the Ministry of Health to allocate dedicated funding across directorates and programs, reserved for managing climate-related health emergencies. Adopting these strategies would significantly improve healthcare financing and enable Sierra Leone to develop a more robust health system well-prepared to confront the challenges posed by climate change.

### Research and data systems

Sierra Leone currently lacks a robust database within its healthcare system to document and monitor the effects of climate change. Moreover, there has been a shortage of research efforts to guide evidence-based solutions on the impact of climate change on the health system. Addressing the health risks associated with climate change remains a significant public health priority. To remedy this, the government should allocate resources to the Ministry of Health to develop a specialized climate change database within the health sector. This effort must be complemented by research initiatives focusing on understanding the long-term consequences of climate change. In addition, the Ministry of Health should partner with research institutions and public health experts to undertake cutting-edge studies on climate-related issues. Securing international collaboration for financial, technical, and logistical support will be pivotal in advancing climate change research in Sierra Leone.

## Conclusions

As Sierra Leone continues to confront emerging public health challenges, including those posed by climate-related risks, it is evident that the impacts of climate change extend beyond healthcare to influence several other sectors, ultimately worsening health outcomes. Given the country's limited resources, addressing the effects of climate change on health systems requires a comprehensive and multifaceted approach. Key challenges such as the lack of climate-resilient infrastructure, workforce shortages, insufficient community engagement in climate-related policy development, inadequate funding, and limited international collaboration must be addressed. Additionally, the absence of robust research initiatives and a reliable data system for monitoring and reporting the health impacts of climate change undermines surveillance efforts and demands urgent attention. By tackling these critical issues, Sierra Leone can develop a climate-resilient health system capable of meeting the population's health needs arising from climate change. This opinion highlights the need for a paradigm shift in Sierra Leone's approach to health systems to ensure a more rapid and effective response to the impacts of climate change on dual disease burden infectious and climate-related diseases.

## Data Availability

Not applicable.

## Abbreviations

NCDs: Non-Communicable Diseases
SDG: Sustainable Development Goal
UHC: Universal Health Coverage
